# Fertilizer Type Affects Stable Isotope Ratios of Nitrogen in Human Blood Plasma—Results from Two-Year Controlled Agricultural Field Trials and a Randomized Crossover Dietary Intervention Study

**DOI:** 10.1021/acs.jafc.1c04418

**Published:** 2022-03-09

**Authors:** Axel Mie, Vlastimil Novak, Mikael Andersson Franko, Susanne Gjedsted Bügel, Kristian Holst Laursen

**Affiliations:** † Department of Clinical Science and Education, Södersjukhuset, Karolinska Institutet, Stockholm 11883, Sweden; ‡ Department of Environmental Science, Stockholm University, Stockholm 106 91, Sweden; § Plant Nutrients and Food Quality Research Group, Plant and Soil Science Section and Copenhagen Plant Science Centre, Department of Plant and Environmental Sciences, University of Copenhagen, Frederiksberg C 1871, Denmark; ∥ Preventive and Clinical Nutrition, Department of Nutrition, Exercise and Sports, University of Copenhagen, Frederiksberg C 1871, Denmark

**Keywords:** dietary protein, fertilizer type, organic food, production system, stable nitrogen isotopes

## Abstract

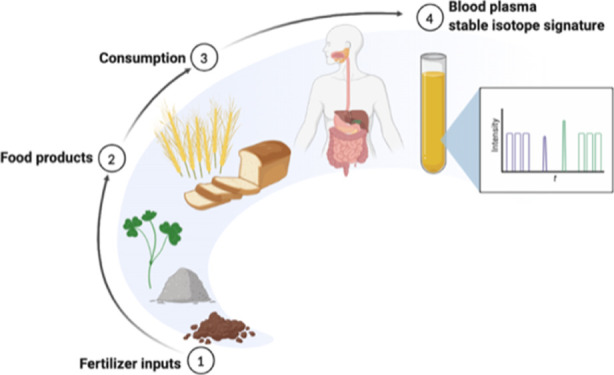

The stable nitrogen isotope ratio δ^15^N is used as a marker of dietary protein sources in blood. Crop fertilization strategies affect δ^15^N in plant foods. In a double-blinded randomized cross-over dietary intervention trial with 33 participants, we quantified the effect of fertilizer type (conventional: synthetic fertilizer and organic: animal or green manure) on δ^15^N in blood plasma. At study baseline, plasma δ^15^N was +9.34 ± 0.29‰ (mean ± standard deviation). After 12 days intervention with a diet based on crops fertilized with animal manure, plasma δ^15^N was shifted by +0.27 ± 0.04‰ (mean ± standard error) compared to synthetic fertilization and by +0.22 ± 0.04‰ compared to fertilization with green manure (both *p* < 0.0001). Accordingly, differences in the δ^15^N values between fertilizers are propagated to the blood plasma of human consumers. The results indicate a need to consider agricultural practices when using δ^15^N as a dietary biomarker.

## Introduction

Stable isotope ratios of light elements are increasingly being used as biomarkers of dietary exposures in nutritional epidemiology, archeology, and ecology.^1^ So far, the stable isotope ratios of hydrogen, carbon, nitrogen, oxygen, and sulfur have attracted the most interest for this purpose. Sulfur isotope ratios (δ^34^S) have the potential to be a useful biomarker of seafood intake, while hydrogen and oxygen isotope ratios (δ^2^H, δ^18^O) of food are affected by the geographic (and climatic) origin but are less developed as dietary biomarkers in human tissue.^1^

Nitrogen has two stable isotopes, ^14^N (light) and ^15^N (heavy), with the light isotope being ∼250 times more abundant on Earth. The isotopic nitrogen composition of a sample (*e.g.*, biological tissue) is conventionally expressed by the δ-notation (δ^15^N), which denotes the heavy to light isotope ratio (^15^N/^14^N) in a sample relative to an international standard (atmospheric nitrogen). Due to isotopic fractionation, the presence of ^15^N and δ^15^N values increases with the trophic level in ecosystems. Consequently, animal-derived foods tend to have higher δ^15^N values than plant-based foods. This relationship is used as a marker of meat consumption in epidemiological and archeological studies.^2^

Agricultural crop production depends on fertilization with nitrogen in the plant-available form (nitrate or ammonium), among other nutrients, and the stable isotope ratio of nitrogen differs between fertilizer types. Synthetic fertilizers produced by the Haber–Bosch process and green manure in the form of leguminous plants both rely on atmospheric nitrogen fixation and their δ^15^N values are close to 0‰, resembling the atmosphere’s isotope signature. Organic fertilizers such as farmyard manure or compost have higher δ^15^N values (clustering around +8‰), due to preferential atmospheric loss of the light nitrogen isotope and isotopic fractionation during biological processes.^3^

Conventional and organic cropping systems generally differ in both the amount and type of applied nitrogen fertilizer. Fertilizer use varies with geographic region, crop, and individual farm, with organic agricultural systems generally relying to a greater degree on farmyard manure and other organically derived fertilizers, such as composts. Consequently, crops from organic systems tend to display higher δ^15^N values than crops from conventional systems, a difference that can be used for organic food authentication.^4,5^ A threshold δ^15^N value of +4.3‰ for organic potatoes has been suggested;^6^ however, this may vary between plant species.

Carbon has two stable isotopes, ^12^C (light) and ^13^C (heavy), and a sample’s stable carbon isotope composition is expressed as δ^13^C relative to an international carbon standard [Vienna Pee Dee Belemnite (VPDB)]. A plant’s δ^13^C value depends not only on its type of photosynthetic physiology (C3 *vs* C4 plants) but also to some degree on local conditions such as nutrient and water availability.^7^

Differences in nitrogen input levels have been shown to affect bulk δ^13^C values of wheat.^8^ Effects of organic *versus* conventional plant production on δ^13^C values have also been observed for some crops,^9,10^ and initial evidence indicates that δ^13^C of certain amino acids of wheat and tomato may be affected by the crop production system.^11,12^ However, differences in δ^13^C values between organic and conventional crops are poorly understood and are much less systematic than observed for δ^15^N.^4^

It is well-established that the primary determinant of stable nitrogen and carbon isotope ratios in human tissues is the diet.^1^ The first hypothesis tested in the present study was that the δ^15^N value in blood plasma of participants in a crossover dietary intervention is affected by the production system and fertilization strategy used for the food that makes up the diet. The second hypothesis tested was that δ^13^C values in blood plasma are affected by the food production system, which was not expected. The overall aim of the study is to unravel if the crop fertilization strategy has a potential to interfere with the use of stable isotope ratios as biomarkers of dietary exposures.

## Materials and Methods

### Production of Crops—Field Trial Design and Sampling

The crops used in the study were produced under field conditions in Denmark in years 2007 and 2008 using three fertilizer treatments: organic production system fertilized with pig manure (OA); organic production system fertilized with legume-based green manures and cover crops, with pig slurry applied to onions and white cabbage to satisfy their high nitrogen demand (OB); and conventional production system relying on inputs of synthetic fertilizers (CON). Potatoes, wheat, barley, rapeseed, and faba beans were grown in the long-term crop rotation experiment CropSys in Foulum, Denmark.^13^ Carrots, onions, white cabbage, and oats were grown in the VegQure rotation experiment in Aarslev, Denmark.^14^ The production systems were established in 2005 (CropSys) or 2006 (VegQure). All crops in all systems were produced in two replicate crop rotations. The organic systems were managed in compliance with the regulations for organic farming in force at that time.^15,16^ Details regarding crop production, soil conditions, climate, plant protection, fertilizer application, yields, and sampling have been reported previously.^17^

### Preparation of Diets

Three diets were tested in the human intervention study: one conventional (CON) and two organic (OA and OB). Each diet was prepared in duplicate according to the crop production replicates described above. The diets included two different menus (Table 1), consumed on alternate days during each intervention period. The menus and the food quantities used in all diets were identical. The content of carbohydrates, protein, and fat in the diets was 52, 15, and 33% of total energy intake, respectively, calculated using the Dankost2000 dietary assessment software (Danish Catering Center, Herlev, Denmark). The portions of the meals were scaled to the estimated energy requirement of each participant prior to the study. A full description of the menus is given in ref (17).

**Table 1:** Composition of the Two Menus Used in the Study

	menu 1	menu 2
breakfast	carrot roll, carrot jam, butter, and skimmed milk	carrot roll, carrot jam, butter, and skimmed milk
lunch	whole-grain bread, meat balls, carrots, barley, and bean salad	whole-grain bread, hummus, and coleslaw salad
dinner	baked potatoes with carrots, minced meat, and vegetables	mashed potatoes and fricassee with beans
snack	carrot cake and oat cookies	potato cake and oat cookies

Protein sources in the intervention diet are shown in Table 2. All major plant-derived ingredients in the intervention diets were produced in the field trials. Animal-derived foods (milk, pork meat, meatballs, eggs, and butter) and food additives (sugar, lemon juice, baking yeast, salt, baking powder, gelatin powder, and pepper) were purchased from local groceries and the same products (irrespective of production system) were included in all intervention diets. Further details regarding preparation of intervention diets have been published previously.^17^

**Table 2:** Protein Sources in the Intervention Diets (g Protein per 10 MJ Menu)

	menu 1	menu 2
legumes	5.70	19.20
cereals (oats, barley, and wheat)	30.27	22.24
other plants (including potatoes)	6.70	8.48
pork (including gelatin)	9.59	0.35
beef	0.56	0
dairy and eggs	17.87	21.02
protein, total	70.69	71.29
plant protein fraction of total protein	60.4%	70.0%
nonleguminous plant protein fraction of total protein	52.3%	43.1%

A 10 MJ portion of each diet (each production system, each field duplicate, each year) was collected, homogenized, freeze-dried (Christ Freeze Dryers, Beta 1–8; Montreal Biotech Inc., Dorval, Canada), and stored in an inert nitrogen atmosphere at −80 °C until analysis for the present work.

### Dietary Intervention Study—Participants

**Figure 1:**
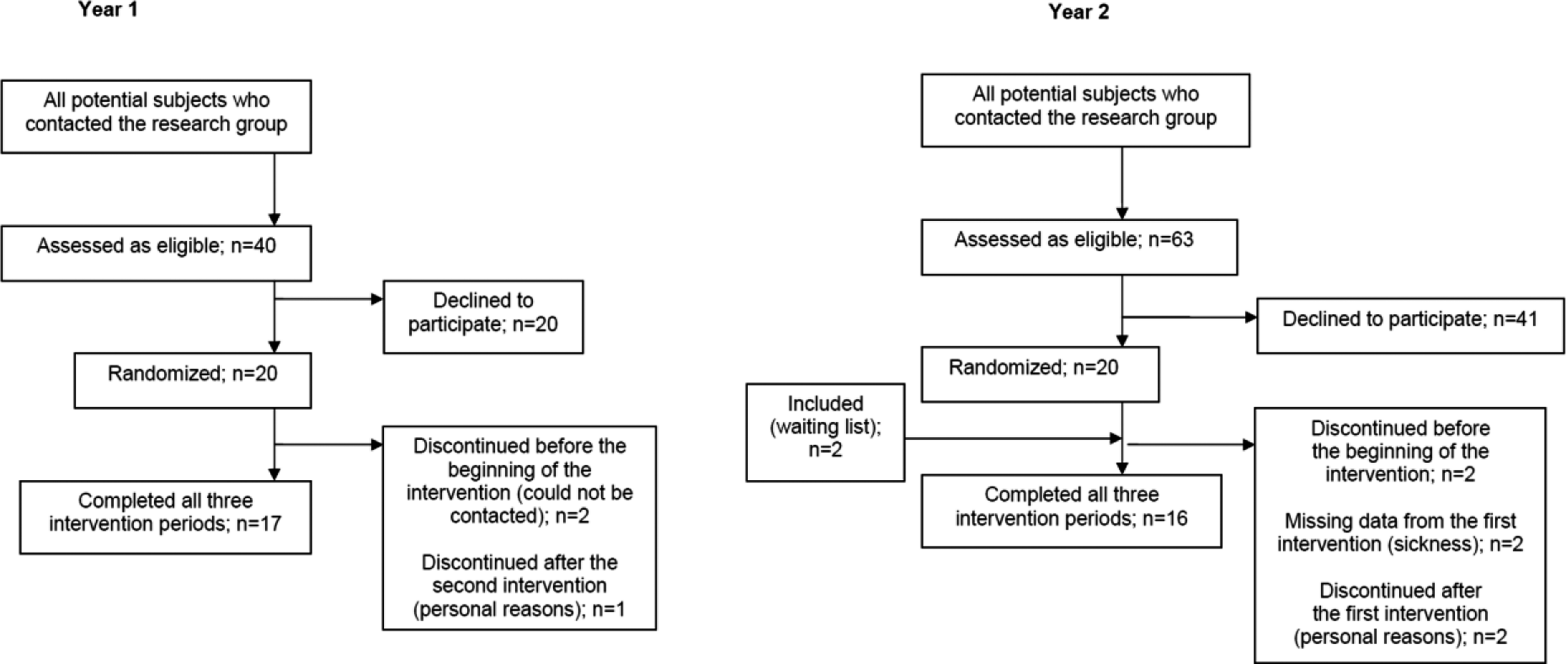
Flowchart of participants in the intervention study in years 1 and 2.

Thirty-three apparently healthy, normal weight, male volunteers completed a double-blinded, crossover, dietary intervention study in one of the two consecutive years [2008 (*n* = 17) and 2009 (*n* = 16)]. The inclusion criteria for participants were age 18–45 years, overall healthy, omnivorous, nonsmokers, and body mass index (BMI; kg m^–2^) < 28. Exclusion criteria were physical exercise more than 10 h per week, alcohol consumption more than 21 units per week, medication, dietary supplement consumption, and blood donation within at least 2 months prior to and during the study. The baseline characteristics for the participants (age, weight, height, and BMI) are reported in ref (17). A participant flow chart is shown in Figure 1.

### Dietary Intervention Study—Design

The double-blinded, crossover, human dietary intervention trial was performed at the Department of Nutrition, Exercise and Sports (previously Department of Human Nutrition), University of Copenhagen (Denmark), from January to April in two consecutive years (2008 and 2009), with diets prepared from the crops harvested in autumn 2007 and 2008, respectively. Hereafter, the year stated refers to the year of harvest. The interventions were performed as three 12-day dietary intervention periods, separated in time by washout periods of a minimum of 14 days. Each volunteer was assigned to one of the six possible sequences of the three interventions by drawing lots to a predefined order to ensure a balanced distribution. A carryover effect was accounted for in the statistical model (see below). The study coordinator was responsible for recruitment, while the principal investigator assigned the participants to the different sequences. The predefined allocation sequence was prepared by the study statistician. The blinding key was broken following the statistical analyses for the original publications from the intervention trial,^17,18^ before the present post-hoc analysis. The kitchen staff prepared and packed each meal for each individual subject in an unblinded fashion. The meals were handed out to the individual participants by the study coordinator. In each year, during each intervention periods 1, 2, and 3, five or six volunteers were given diets produced from CON, OA, and OB crops. Energy requirement for each individual subject was calculated from reported physical activity, weight, and age prior to the beginning of the study. Details about the study design are reported in ref (17).

This dietary intervention study was registered at http://www.clinicaltrials.gov (NCT00738166). The primary outcome measure of the study was the bioavailability of trace elements and secondary metabolites, as reported previously.^17,18^

### Blood Samples

Baseline blood samples were collected in the morning (07.30–09.00) on day 1 and end-time blood samples were taken in the morning after the last meal (day 13) of each dietary period. Fasting blood samples were drawn from a forearm vein puncture into EDTA tubes (Hemogard Plus 10 mL K_2_EDTA tubes, Becton Dickinson, Kongens Lyngby, Denmark), centrifuged at 2200*g* and 4 °C for 15 min, and stored at −80 °C until analysis. The subjects were instructed to fast (>12 h) and to avoid alcohol and strenuous exercise for 48 h before blood sampling.

### Stable Isotope Ratio Analysis of Fertilizers, Crops, and Diets

The δ^13^C and δ^15^N analysis of crops and diets was conducted using a Europa Scientific ANCA-SL elemental analyzer coupled to a Europa Scientific 20–20 Tracermass mass spectrometer (Sercon Ltd., Crewe, UK), using 4 mg of plant/diet powder packed in tin capsules as detailed in ref (10). The nitrogen isotope composition of the fertilizer samples was determined using a Costech elemental analyzer coupled to a Thermo Finnigan Delta XP continuous flow IRMS (Thermo Scientific, Bremen, Germany).

For each crop, 12 samples were analyzed: one sample per production system, field replicate, and year. For 2007, whole diet (pooled day 1 + day 2 menus) freeze-dried samples were analyzed in technical duplicate for each production system and field replicate (12 analyses). For 2008, day 1 and day 2 diets were analyzed separately in technical duplicate for each production system and field replicate (24 analyses).

For several crops, certain stable isotope data from the field trials have been published previously, as specified in Table 3.

**Table 3:** Mean Stable Isotope Ratios by Crop, Year, and Production System^a^

	δ^15^N			δ^13^C		
	means in ‰			means in ‰		
	CON	OA	OB	CON	OA	OB
Faba Beans						
2007	0.8	0.6	0.4	–27.5	–27.8	–27.8
2008	–0.3	1.0	0.3	–25.8	–25.7	–25.9
Wheat						
2007	1.2	7.2	0.8	–26.1	–25.6	–24.7
2008	1.4	7.4	1.8	–25.5	–24.8	–24.0
Barley						
2007	1.4	5.3	0.5	–27.3	–27.2	–27.4
2008	1.9	5.3	0.8	–25.9	–26.2	–26.5
Rapeseed						
2007	0.4	7.2	2.6	–27.9	–28.2	–29.2
2008	4.2	2.4	8.8	–28.5	–27.4	–26.8
Potatoes						
2007	0.8	3.0	0.9	–28.1	–26.6	–26.2
2008	0.2	4.1	1.4	–27.2	–25.6	–25.2
Oat						
2007	1.4	6.0	5.2	–28.9	–29.0	–28.7
2008	4.0	6.1	3.2	–24.8	–25.2	–26.1
Carrots						
2007	3.0	3.5	3.5	–27.9	–28.2	–28.3
2008	2.0	5.6	5.1	–27.5	–27.5	–27.3
Cabbage						
2007	4.2	7.8	3.9	–24.1	–24.1	–23.9
2008	2.3	7.8	4.8	–24.0	–23.6	–23.5
Onions						
2007	1.4	4.8	6.3	–26.8	–27.8	–27.2
2008	0.7	6.4	6.9	–24.9	–25.3	–25.5

a Due to the small number of samples (*N* = 4 per year, crop, and production system), no standard deviations are shown. CON = conventional, OA and OB = organic fertilized with pig manure and legumes, respectively. Certain results presented in this table for completeness have been published earlier: Faba beans, barley, and potatoes: refs (10, 9) (potatoes) report averages for three locations. Wheat: ref (10) reports these values for 2007. Carrots and cabbage: ref (9) reports averages for all three field replicates. For 2008, rapeseed shows a deviating pattern from other crops with respect to δ^15^N, possibly due to a mistake at some point in the fertilization, harvesting, sampling, and analysis chain. As rapeseed oil contributes only trace amounts of nitrogen to the diets, this possible mistake could not have affected the δ^15^N in analyses of diets and human plasma.

### Stable Isotope Ratio Analysis of Human Blood Plasma

Prior to analysis, 7 μL of blood plasma was transferred to a tin capsule and left to evaporate at room temperature for 12 h. The samples were fully dried in a rotational vacuum concentrator (Martin Christ Freeze Dryers, Osterode, Germany) set to 30 °C for 1 h. The samples were then tightly packed into their tin capsule and one additional tin capsule, to avoid sample loss. This sample preparation was developed from the method reported by Patel *et al.* 2016.^19^

Stable isotope ratio analyses (δ^13^C and δ^15^N) of dried human plasma were conducted using a PYRO Cube elemental analyzer (EA) (Elementar, Hanau, Germany) coupled to an Isoprime100 stable isotope ratio mass spectrometer (Elementar, Manchester, UK). The PYRO Cube EA combustion tube was set to 1120 °C, reduction tube to 850 °C, oxygen flow to 10 mL/min, and helium flow to 230 mL/min. Raw isotope data were corrected for instrument drift and linearity and calibrated to international scales using the following procedures: A correction for instrument drift over time was achieved by repeated analysis of nylon (calibrated against Standard Reference Materials IAEA-600, USGS-40, USGS-41, USGS-42, USGS-43, USGS-61, USGS-64, and USGS-65, obtained from the Stable Isotope Facility at UC Davis, California, USA) at every 14th position in the sample sequence and calculated as dependence of the measured isotope value on sample position. The linearity effect was evaluated by inclusion of six samples of acetanilide (Sigma-Aldrich) in decreasing amounts at the beginning and at the end of the sequence (∼1700–150 μg C and ∼250–20 μg N). The linearity correction factor was calculated as a linear function of peak area and measured raw isotope values. The correction factor was negligible for nitrogen, and δ^15^N values were not corrected for linearity. Accuracy of the analysis was assured by a final two-point calibration using United States Geological Survey l-glutamic acid reference isotope standards USGS 40 (δ^15^N = −4.52; δ^13^C = −26.39) and USGS 41 (δ^15^N = 47.57; δ^13^C = 37.63), which were purchased from the International Atomic Energy Agency (IAEA, Vienna, Austria). Precision was calculated using 7 μL aliquots of an in-house blood plasma standard prepared as described above and included at every 14th position in the sample sequence. Samples were analyzed in two batches, one for each year. Precision in batch 1 and 2 was 9.19 ± 0.16‰ [mean ± standard deviation (SD)] and 9.31 ± 0.06‰, respectively, for δ^15^N, and −23.97 ± 0.02 and −24.01 ± 0.02‰, respectively, for δ^13^C, indicating high reproducibility of the analytical platform within and across batches.

Isotope values were reported using conventional δ-notation as
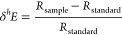
1where *h* is the heavy isotope of an element E (*e.g.*, ^15^N or ^13^C) and *R* is the corresponding ratio of heavy/light isotopes (*e.g.*, ^15^N/^14^N or ^13^C/^12^C). The δ-notation isotope values were reported in parts per thousand (‰) with respect to the international standards (*R*_standard_) air for δ^15^N and Vienna Pee Dee Belemnite (VPDB) for δ^13^C.

### Statistical Analysis

All analyses in this paper are regarded as explorative, as the hypotheses had not been included when the primary and secondary outcomes were defined during the design of the dietary intervention study.

Linear mixed models were used to model the stable isotope composition of crops, diets, and human plasma. Fixed factors and interactions as well as random factors included were identified based on the knowledge of the design and data structure of the experiments. The structure of the variance-covariance matrix was determined using the knowledge of the study design, Akaike information criterion (AIC), and Bayesian information criterion (BIC). Additional details on the development of the linear mixed models are reported in the Supporting Information.

Linear mixed models were built in R software version 4.0.0^20^ using the package “nlme” version 3.1-147.^21^ Overall *p*-values were derived from an ANOVA of Type II sums of squares using the Anova() function from the R package “car”.^22^ Estimated effect sizes and p-values from t-tests for pairwise comparisons within mixed models were derived from Wald tests using the summary() function. All p-values are reported as unadjusted for multiple testing.

Crops: Three main effects, production system (CON, OA, and OB), year (2007 and 2008), and field replicate (1 and 2), without interactions, were used to model crop stable isotope ratios. Crop type was specified as a random effect.

Intervention diets: Four fixed effects, system, year, field replicate, and menu, without interactions, were used to model the stable isotope composition of the intervention diets. Sample ID was specified as random factor, to account for the technical-analytical duplicate.

Blood plasma: Five fixed effects, namely, system, year, field replicate, day (0 and 12), and carryover, and the interaction (system * day), were used to model the stable isotope composition in blood plasma. As a random factor, the subject (“subject”) was included. The covariance structure of the final model was continuous autoregressive of order 1 [CAR(1)].

### Kinetics of Stable Nitrogen Isotopes in Human Blood Plasma

A previous feeding study in black bears demonstrated, over a wide range of dietary δ^15^N (+2.5 to +11‰), that a change in dietary δ^15^N translates into an equally sized change in blood plasma δ^15^N, given sufficient time for reaching equilibrium.^23^ Visual inspection of the time series in a human intervention study indicated that first-order kinetics could be a reasonable initial approximation for stable nitrogen isotope turnover in plasma.^24^

Assuming a simple first-order kinetic model, we estimated the kinetics of δ^15^N in blood plasma using the difference in δ^15^N in a pair of diets (CON and OA) and in the corresponding blood plasma samples after 12 days of dietary intervention, according to the exponential decay function
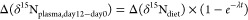
2with half-life
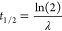
3where Δ(δ^15^N_plasma_,_day12–day0_) represents the effect of OA *versus* CON diets on the plasma δ^15^N after 12 days of intervention, that is, the effect size for the (system *x* day) interaction for production system OA compared with CON (Table 4); Δ(δ^15^N_diet_) represents the difference in the stable nitrogen isotope ratio of the diets from system OA compared with CON (Table 4); λ represents the incorporation rate constant (or decay constant); *t* is the length of the intervention (12 days); and *t*_1/2_ is the half-life of stable isotope turnover in plasma.

### Ethical Permit

The dietary intervention study was approved by the Danish National Committee on Biomedical Research Ethics of the Capital Region of Denmark (J. no. H-C-2007-0078). The present analysis of stable isotopes in blood plasma samples from that study was later approved (J. no. H-15016596) (Jan 19, 2016) by the same committee. The procedures followed the ethical standards of this committee.

## Results

### Fertilizers

Small numbers of fertilizer samples were tested for their stable nitrogen isotope composition. The δ^15^N values of three samples of synthetic fertilizers used at the trial sites in Aarslev and Foulum in 2008 averaged at −0.4‰ (range −0.8, +0.4). Two samples of animal manure from the study sites, taken in 2008, had δ^15^N values of 4.2 and 12.5‰, respectively. Although the number of samples was small, means and ranges were consistent with δ^15^N values in fertilizers reported previously.^3^

### Crops

Table 3 shows mean stable isotope composition of crops for those field locations and field replicates which supplied the ingredients for the intervention diets. Overall, as indicated by the statistical analysis shown in Table 4, crops from the pig manure-fertilized OA system generally had higher δ^15^N values than crops from the CON and OB systems, which were fertilized with synthetic N and leguminous cover crops, respectively. We observed no apparent effect of the production system on the δ^15^N of the leguminous crop faba bean, which sources its nitrogen from the atmosphere *via* biological nitrogen fixation. The δ^15^N of all the remaining crops was apparently affected. The highest δ^15^N values for the OA system were observed for wheat and cabbage, which is likely due to their high nitrogen demand. Less systematic differences between cropping systems were observed for low nitrogen-demanding crops such as carrots. On average across all nonleguminous crops, the δ^15^N of samples from the OA system was shifted by +3.2 ± 0.3‰ (mean ± SD) compared with samples from the CON system and by +2.6 ± 0.3‰ compared with samples from the OB system. There was no apparent effect of the production year on δ^15^N among the nine crops (Supporting Information Table S1).

**Table 4:** Effect Sizes and *p*-Values for Linear Mixed Models Describing the Effect of the Production System on Stable Isotope Composition of Crops, Diets, and Blood Plasma^a^

	δ^15^N		δ^13^C	
	effect size (‰) mean ± SE	*p*-value	effect size (‰) mean ± SE	*p*-value
Crops^b^				
system		<0.0001		0.96
OA *vs* CON	+3.5 ± 0.3	<0.0001	+0.03 ± 0.10	0.77
OB *vs* CON	+0.5 ± 0.3	0.050	+0.01 ± 0.10	0.90
OA *vs* OB	+2.9 ± 0.3	<0.0001	–0.02 ± 0.10	0.87
Diets				
system		<0.0001		0.12
OA *vs* CON	+1.5 ± 0.2	<0.0001	+0.04 ± 0.07	0.57
OB *vs* CON	+0.1 ± 0.2	0.56	+0.15 ± 0.07	0.070
OA *vs* OB	+1.3 ± 0.2	<0.0001	–0.10 ± 0.07	0.18
Plasma				
system * day interaction (difference of shift day 12–day 0)		<0.0001		0.0079
OA *vs* CON	+0.27 ± 0.04	<0.0001	+0.04 ± 0.03	0.24
OB *vs* CON	+0.05 ± 0.04	0.27	+0.10 ± 0.03	0.0024
OA *vs* OB	+0.22 ± 0.04	<0.0001	–0.06 ± 0.03	0.060

a Fixed factors: production system, year, field replicate, menu (diet only), day (plasma only), system * day interaction (plasma only), and carryover (plasma only). Random factors: crop type (crops), menu (diets), and subject (plasma). Additional parameter estimates and tests are reported in the Supporting Information. SE = standard error. CON = conventional, and OA and OB = organic fertilized with pig manure and legumes, respectively.

b δ^15^N: all crops except faba beans included. δ^13^C: all crops included.

For δ^13^C, no consistent effect of the production system was noted, although there was a numerical difference for some crops (wheat and potatoes) (Table 3). Consistently, across crops, production year affected the δ^13^C values (Supporting Information Table S1).

### Diets

The observed stable isotope composition of whole intervention diets is reported in Table 5. As expected from the ingredient crops, the production system had a clear effect on the δ^15^N of intervention diets. On average, the δ^15^N of OA diets was +1.5 ± 0.2‰ higher than that of CON diets and +1.3 ± 0.2‰ higher than that of OB diets (mean ± SE). The magnitude of these shifts is approximately half that of the corresponding δ^15^N shift between systems for crops (Table 4). This is explained by the fact that only approximately half the protein in the intervention diets can have been affected by the production system, as leguminous plants are not affected, and animal-derived proteins were from externally sourced identical ingredients for all diets (see Table 2). Similar to the ingredient crops, there was no apparent effect of production year or of field replicate on δ^15^N of diets (Supporting Information Table S2).

**Table 5:** Stable Isotope Composition of Intervention Diets^a^

	‰, means					
	δ^15^N			δ^13^C		
	CON	OA	OB	CON	OA	OB
2007						
menu 1 + 2	2.1	3.6	2.8	–27.0	–27.1	–26.9
2008						
menu 1	3.0	4.7	2.8	–26.2	–26.0	–26.0
menu 2	1.9	3.1	1.8	–26.4	–26.3	–26.2
2007 + 2008						
overall mean	2.3	3.8	2.6	–26.6	–26.6	–26.5

a Each value represents the mean of two analytical-technical replicates of one sample each from field replicates 1 and 2 (*i.e., N* = 4). For 2007 diets, composite samples of menus 1 + 2 were collected for analysis. The standard deviation for analytical-technical replicates was 0.31‰ for δ^15^N and 0.12‰ for δ^13^C, assuming independence across years, production systems, and menus. (CON = conventional and OA and OB = organic fertilized with pig manure and legumes, respectively)

Finally, menu 1 had a higher δ^15^N value than menu 2, due to the higher fraction of animal-derived proteins in menu 1 and legume-derived proteins in menu 2 (Table 2).

The production system had no pronounced effect on the δ^13^C of the intervention diets, as expected from the analysis of single crops (Table 4).

There was a pronounced effect of production year on δ^13^C, as expected from the carbon isotope composition of the ingredient crops (Supporting Information Table S2). Additionally, menu 2 had slightly lower δ^13^C than menu 1 (Table 5), which can be attributed to the different composition of the two menus and possibly to the higher fraction of animal-derived ingredients in menu 1 (Table 1).

### Plasma

Population mean and standard deviation of the stable isotope ratios for δ^15^N and δ^13^C in blood plasma at study baseline (at day 0 of the first dietary intervention period) are shown in Table 6. For δ^15^N, there was no apparent effect of production year (*t*-test *p* = 0.43). For δ^13^C, a trend for a higher value in 2008 was indicated but not certain (*t*-test *p* = 0.082).

**Table 6:** Observed Population Means and Standard Deviations of Plasma Stable Isotope Ratios at Baseline (Day 0 of the First Intervention) and at Day 12 (*i.e.*, at the End) of Each Intervention Phase^a^

	δ^15^N				δ^13^C				
	means ± SD in ‰				means ± SD in ‰				
	baseline	CON	OA	OB	Baseline	CON	OA	OB	*N*
2007	9.30 ± 0.34	9.04 ± 0.29	9.26 ± 0.27	9.10 ± 0.29	–23.10 ± 0.33	–23.61 ± 0.24	–23.52 ± 0.28	–23.56 ± 0.25	17
2008	9.38 ± 0.22	9.17 ± 0.25	9.38 ± 0.22	9.23 ± 0.28	–22.91 ± 0.28	–23.23 ± 0.20	–23.18 ± 0.21	–23.17 ± 0.20	16
2007/08	9.34 ± 0.29				–23.00 ± 0.32				33

a The range at baseline was 8.36 to 9.96‰ (δ^15^N) and −23.91 to –22.25‰ (δ^13^C). CON = conventional and OA and OB = organic fertilized with pig manure and legumes, respectively. SD = standard deviation.

Observed mean plasma stable isotope ratios for the study population at the end of each intervention phase are shown in Table 6.

The effect of most interest in the present work was the effect of production system on the δ^15^N shift in day 12 compared with day 0 samples, that is, the differential effect of the dietary interventions on plasma δ^15^N. This effect was represented by the interaction term (system * day) in the statistical model (Table 4). The results showed that the plasma δ^15^N at the end of the dietary intervention (day 12) relative to the beginning (day 0) was shifted by +0.27 ± 0.04‰ [mean ± standard error (SE)] on consuming an OA diet compared with a CON diet. The corresponding shift for OA compared with OB consumption was +0.22 ± 0.04‰. The direction of this effect, and also the slightly more pronounced effect in OA *versus* CON compared with OA *versus* OB consumption, was well-preserved from the diets and from the individual crops (Table 4). The magnitude of effect of production system on plasma δ^15^N was smaller than the corresponding difference between diets. This was because a 12-day dietary intervention is too short for full turnover of plasma nitrogen, an aspect further addressed below, in the section “Kinetics”.

Year, field replicate, and production system (reference day 0) had no apparent effect on plasma δ^15^N. Sampling day had a pronounced effect, with plasma δ^15^N decreasing for systems CON and OB but increasing for OA, when comparing day 12 with day 0 (Supporting Information Table S3). As conventional food is the dominant food consumed in Denmark at the population level, this suggests that the habitual diet of participants before the study or during washout periods was generally higher in δ^15^N than the intervention diets possibly because it contained more animal-derived proteins.

There was a significant carryover effect, indicating that the washout periods were not sufficiently long in order to restore plasma δ^15^N to study baseline values.

For δ^13^C, a slight effect of production system on the shift in δ^13^C on day 12 compared with day 0 was indicated (Table 4). The magnitude of this effect was comparable to the differences in δ^13^C between diets but substantially smaller than the effect of production year or of intervention *versus* habitual diet (Supporting Information Table S3). A similar effect was indicated in the whole diets and in two of the major carbon sources in the diets (potatoes and wheat).

Production year substantially affected plasma δ^13^C, with higher values in 2008 than in 2007, which is consistent with the corresponding observations in crops and diets. An apparent effect of field replicate on plasma δ^13^C was surprising, given the absence of a corresponding effect in crops and diets. Further analysis revealed that at study baseline, participants receiving diets from field replicate 2 had lower plasma δ^13^C [−0.25 ± 0.10‰ (mean ± SE), *p* = 0.022] than participants receiving diets from replicate 1. This coincidental difference could plausibly have mimicked an apparent effect of field replicate on plasma δ^13^C and was therefore not considered real. There was no main effect of production system on plasma δ^13^C, with day 0 as reference (Supporting Information Table S3).

Detection of a main effect of day of sampling, similar to all diets, indicates that the intervention diet had lower δ^13^C than the participants’ habitual diets. This likely reflects a comparatively low fraction of animal-derived foods in the intervention diets. Finally, there was a slight carryover effect, indicating that plasma δ^13^C was not restored to baseline values during washout periods (Supporting Information Table S3).

### Kinetics of Stable Nitrogen Isotopes in Human Blood Plasma

The OrgTrace dietary intervention study was not designed for determining the kinetics of stable isotope turnover in blood plasma. Given the scarcity of such studies in humans,^1,24^ the present data were nonetheless used to derive an estimate of the half-life of δ^15^N in human plasma and thus of the temporal window of dietary exposure that is captured by stable nitrogen isotopes in plasma.

The largest pairwise δ^15^N difference between intervention diets [+1.47 ± 0.19‰ (mean ± SE); Table 4] was observed for system OA compared with CON. This pair was selected for further calculations. The corresponding shift in plasma δ^15^N between the OA and CON diets after 12 days of dietary intervention was only +0.27 ± 0.045‰, indicating that the intervention periods were too short to establish a new equilibrium. Assuming a first-order kinetic model for reaching stable N isotope equilibrium in blood plasma after a dietary change, the point estimate for the fractional incorporation rate is λ = 0.0168 day^–1^ [95% confidence interval (CI) 0.00937–0.0248]. The mean half-life of δ^15^N in human plasma was estimated to be 41 days (95% CI 28–74), suggesting that plasma δ^15^N reflects the diet of the last few months. It must be stressed that this calculation constitutes an initial estimate, which may inform but not replace a dedicated study.

For δ^13^C, a meaningful estimate for half-life in plasma could not be derived because the differences between production systems were too small and uncertain.

## Discussion

### Conservation of Stable Nitrogen Isotope Ratios across the Study

This study demonstrated that the fertilization strategy applied in crop production leaves a stable nitrogen isotope signature in crops and diets, which is then measurable in blood plasma of human consumers. To our knowledge, this is the first study to couple a controlled agricultural field trial using different fertilization strategies for food production with a clinical dietary intervention trial, in order to track stable isotope ratios from agricultural crop management to human blood plasma.

Specifically, this study demonstrated that consumers of a diet produced using pig manure as fertilizer had a stable nitrogen isotope ratio in plasma that was shifted substantially compared to consumers of a diet produced from crops fertilized with nitrogen fixed from the atmosphere (synthetic fertilizer or green manure). This was shown using crops produced two to four years after the establishment of the different production systems on the field sites.

Characteristically, organic production relies on animal manure to a higher degree than conventional production. The present results suggest that the stable nitrogen isotope ratio in blood plasma is potentially increased in consumers with a high fraction of organically produced products in the diet. The potential scope, limitations, and possible future developments of this finding are discussed below.

As expected, no systematic effect of the agricultural production system on stable carbon isotope values of crops, diets, and plasma was identified in this study. Therefore, δ^13^C is not further discussed below.

### Can δ^15^N Be a Biomarker for Organic Food Consumption?

A small number of large prospective cohort studies have reported associations between organic food consumption and health outcomes.^25−29^ These studies all rely on self-reported frequencies of organic food consumption for assessment of exposure. In the Million Women study in the UK, consumption of organic food was associated with a decreased risk of developing non-Hodgkins lymphoma. Recently, similar findings were reported for the Nutrinet-Santé study.^29^ The potential importance of this association for public health warrants the development of tools for assessing organic food consumption more reliably.^30^ A particular complication is a risk of response bias, as health-conscious study participants may overestimate their organic food intake while also favoring other positive health behaviors.^30^ A biomarker of organic food consumption that is not prone to response bias would advance the methodology for research on human health effects of organic production methods.

Fertilization in both organic and conventional production varies with region, crop, and farm. Still, crop δ^15^N is consistently reported to be elevated in organic compared with conventional crops across regions.^31^ Despite local and regional variations in plant nitrogen sources, it is plausible to assume that at a large scale, organic plant foods generally have elevated δ^15^N values compared with conventional plant foods, due to differences in the fertilization regime.

Differences in habitual protein sources in the present study led to a range of plasma δ^15^N values at study baseline spanning 1.6‰ (Table 6). For comparison, given sufficient time, a change in plasma δ^15^N of +1.5‰ would be expected for organic farmyard manure-fertilized diets compared with conventional synthetically fertilized diets in the present study. Under real-life conditions, the difference between production systems would be expected to be substantially smaller, due to the common use of nitrogen-fixating plants in organic farming and the use of animal manure in conventional farming.

It is obvious from the abovementioned discussion that plasma δ^15^N cannot be used as a standalone biomarker of organic food intake, without taking dietary habits into account. Data on the proportion of dietary proteins in food items or food categories are commonly available from validated food records in nutritional-epidemiological studies. If plasma δ^15^N is adjusted for dietary protein sources, its variation due to the production system may potentially be sufficiently high to reflect the fraction of organic food in the diet. It must be kept in mind that this would not measure the organic fraction of all food consumed, but rather the organic fraction of nonleguminous plant protein consumed, although these two quantities might be well-correlated. For example, plasma δ^15^N may contribute to a validation of questionnaires regarding organic food consumption. The present lack of such validation is a limitation in epidemiological studies addressing potential health effects of organic food.

### Implications for Epidemiological and Archeological Studies

As with any nutritional epidemiology study, the level of confidence in exposure assessment increases with the use of validated biomarkers compared with self-reported dietary records because biomarkers are not prone to reporting biases. Although not yet routinely used at a large scale, stable isotope ratios carry the promise of providing biomarkers of exposure for nutritional epidemiology. Most notably so far, δ^15^N has been used as a marker of meat consumption in epidemiological studies.

It has recently been suggested that organic food consumption should not be overlooked in such cases.^32^ The recent and ongoing increases in organically managed agricultural land and in market shares of organic products in Europe, USA, China, and other regions create a need to understand whether and how the production system interferes with other established dietary biomarkers.^32^

Both meat consumption and organic food consumption are positively associated with plasma δ^15^N. There are indications that meat consumption is positively and organic food consumption is negatively associated with certain health outcomes, specifically non-Hodgkins lymphoma^25,29,33^ and type 2 diabetes/metabolic syndrome.^34−36^ In populations with substantial meat and organic food consumption, this may create a situation of negative confounding: any potential associations between δ^15^N and disease risk may plausibly be interpreted differently if δ^15^N is used as a marker of either meat consumption or organic food consumption. Conversely, in conjunction with additional high-quality information on dietary protein sources, for example, from validated dietary questionnaires, plasma δ^15^N could be helpful in disentangling the effects of these opposing factors on disease risk.

In archeology, δ^15^N in prehistoric human bone collagen is often used as a marker of meat consumption, where higher δ^15^N values indicate a higher fraction of animal-based foods in the diet. However, it has been recognized that nutrient cycling with human or animal manure in prehistoric agricultural societies increases dietary δ^15^N independently of meat consumption,^37^ as also indicated in the present work. Accordingly, bone collagen δ^15^N may represent a marker of meat consumption or a marker of food production system or a combination of both.

### Perspectives

Based on the present work, we propose that the effect of organic *versus* conventional food consumption on plasma δ^15^N values warrants further evaluation in a human population consuming habitual diets. This evaluation should clarify if organic food preference may interfere with the use of δ^15^N in blood as a biomarker for dietary protein sources. This evaluation should further evaluate under which conditions, if any, plasma δ^15^N may be used to assess or confirm reported consumption of organic food.

A dedicated study on the kinetics of human-stable nitrogen isotope turnover in response to dietary changes would be helpful for establishing the time scale of dietary habits covered by δ^15^N in plasma and possibly in other tissues. For plasma, our estimate for the mean incorporation rate constant of λ = 0.118 week^–1^ (95% CI 0.0656, 0.174) based on 33 participants corresponds to a previously reported median λ = 0.19 week^–1^ (interquartile range 0.13, 0.36) based on 15 participants with converging δ^15^N out of a total of 32 participants,^24^ with a point estimate for a half-life of 5.9 and 3.7 weeks, respectively. Plasma δ^15^N would accordingly reflect dietary exposure during previous months. Both estimates were explicitly described as preliminary. Initial evidence suggests that urinary urea reflects the dietary δ^15^N of the last few meals in humans,^38^ while red blood cells would be expected to reflect δ^15^N during their turnover time of 3–4 months. Also, human hair δ^15^N has been demonstrated to change in response to a dietary intervention and offer an opportunity for time-resolved measurements.^39^ As in other species, δ^15^N in different human tissues may be used for assessing exposure during different temporal windows.^40^ This could potentially be complemented by nontraditional isotopes as recently documented for sulfur, strontium, lead, zinc, iron, copper, magnesium, and cadmium in hair and nails.^41^ Compound-specific stable isotope ratio analysis, where isotopes are measured in specific compounds such as amino acids rather than in bulk plasma, potentially offers additional specificity in measuring dietary protein sources, both with respect to the production system^11^ and type of protein.^42^ While the techniques for compound-specific analysis of stable isotopes are currently too laborious and costly for large-scale use in epidemiological studies, this might change in the future.

## Supporting Information Available

Additional details regarding the statistical methods; detailed results for crop-stable isotope composition in relation to the production system and other factors; detailed results for diet stable isotope composition in relation to the production system and other factors; and detailed results for plasma-stable isotope composition in relation to the production system and other factors (PDF)

## Abbreviations

AIC: Akaike information criterion
AR(1): first-order autoregressive covariance structure
BIC: Bayesian information criterion
CON: conventional production system fertilized with mineral (synthetic) fertilizer
CAR(1): first-order continuous autoregressive covariance structure
CI: confidence interval
CS: compound symmetry covariance structure
δ^13^C: stable carbon isotope ratio
δ^15^N: stable nitrogen isotope ratio
IAEA: International Atomic Energy Agency
λ: incorporation rate constant (or decay constant)
OA: organic production system fertilized with pig manure
OB: organic production system fertilized with legume-based green manures and cover crops
*R*: ratio of heavy/light isotopes
SD: standard deviation
SE: standard error
*t*_1/2_: half-life
UN: unstructured covariance structure
USGS: United States Geological Survey
VPDB: Vienna Pee Dee Belemnite
