# Supplementary material for: Fertilizer Type Affects Stable Isotope Ratios of Nitrogen in Human Blood Plasma—Results from Two-Year Controlled Agricultural Field Trials and a Randomized Crossover Dietary Intervention Study
Source: J Agric Food Chem. 2022 Mar 9;70(11):3391–9. doi: 10.1021/acs.jafc.1c04418 (PMC8949720; doi:10.1021/acs.jafc.1c04418)
Supplement: Supplementary file 1 — jf1c04418_si_001.pdf [file jf1c04418_si_001.pdf]

Supplementary file to:

Fertilizer type affects stable isotope ratios of nitrogen in human blood plasma – results from two-year controlled agricultural field trials and a randomized cross-over dietary intervention study

Axel Mie, Vlastimil Novak, Mikael Andersson Franko, Susanne Gjedsted Bügel, Kristian Holst Laursen

This file contains additonal details regarding statistical methods and results

Methods

A priori, the variance was assumed to be homogenous and the most suitable covariance structure considering the study design was chosen. If applicable, alternative structures, specifically heterogeneous variance as well as a simpler and a more complex covariance structure, were evaluated and chosen if they led to a decrease in AIC of at least two units compared with the *a priori* expected model. Alternative structures were avoided if they led to a substantial increase in BIC with increased model complexity, which was interpreted as a sign of overfitting [19-21].

Model assumptions were evaluated as follows: fitted values were plotted against standardized residuals and the plot was visually examined for extreme values (outliers) and structural elements not accounted for in the model. Normal distribution of residuals (error terms and random effects) was evaluated by visual inspection of quantile-quantile plots and Shapiro-Wilks test. Data transformations (inverse, square root, log transformation, inverse of square root) were made when a deviation from normality was indicated. Correlation between random effects and error terms was

evaluated by plotting and by calculating and testing Pearson's correlation. The source of any deviation was investigated and addressed in a sensitivity analysis; results from the modified model were compared with those of the original model, and any major deviations reported. If no major deviations were observed, only the results from the main model representing the entire study population were reported.

Crops: Three main effects, production system (CON, OA, OB), year (2007, 2008), and field replicate (1, 2), without interactions, were used to model crop stable isotope ratios. Crop type was specified as a random effect, which implies that the crops included were regarded as representing a larger population of crops that could potentially be included in diets. For $\delta^{15}N$, faba beans were excluded from this model due to a different mode of nitrogen uptake compared with all other crops included (atmospheric fixation compared with uptake from soil). For $\delta^{15}N$ and $\delta^{13}C$, heterogeneity of variance between crops was included in the statistical model.

Intervention diets: Four fixed effects, system, year, field replicate, and menu, without interactions, were used to model the stable isotope composition of the intervention diets. For 2007, pooled samples of menu 1 and 2 were analyzed, so menu was included as a numerical covariate with a value of 1 for menu 1 (in 2008), 2 for menu 2 (in 2008), and 1.5 for pooled menus (in 2007). Sample ID was specified as random factor, to account for the technical-analytical duplicate.

Blood plasma: Five fixed effects, i.e., system, year, field replicate, day (0, 12), and carryover, and the interaction (system * day), were used to model the stable isotope composition in blood plasma. Carryover was a composite factor with seven levels, describing the position of the intervention in the sequence (1st, 2nd, or 3rd) and, for the 2nd and 3rd intervention, the production system of the preceding intervention. The interaction (system * day) represents the effect of the production system of the diet

on the shift in the plasma stable isotope composition from day 0 to day 12. A test of this interaction corresponds to a test of the main hypothesis of this study.

Due to an expected autoregressive element in the data and varying length of washout periods, a first-order continuous autoregressive (CAR(1)) covariance structure with constant variances was expected *a priori* to best describe the data. A covariate representing the chronological time since study baseline (“chron”) was included in the model for fitting the CAR(1) covariance structure. This structure was compared with three alternative structures: compound symmetry (CS), first-order autoregressive (AR(1)), and unstructured (UN).

Heterogeneous variances with respect to day were considered; plausibly, the variance could be smaller on day 12 compared to day 0, because study participants consumed more similar diets during the 12-day intervention than habitually or during washout periods. All combinations of covariance and variance structures were considered.

As random factor, the subject (“subject”) was included.

For $\delta^{15}N$, the selected covariance structure was CAR(1) with homogeneous variances. Plotting fitted values against standardized residuals clearly identified one outlying subject. All model assumptions tested were fulfilled upon removal of this subject from the model. This removal had only minor effects on the estimates of coefficients and on statistical significance.

For $\delta^{13}C$, the selected covariance structure was CAR(1) with heterogeneous variances with respect to day. A deviation from normal distribution of error terms was indicated, apparently due to day 0 values for certain subjects. A slight negative correlation between random effects and error terms was also indicated (r=-0.15,

p=0.041). Removing five subjects with clearly outlying data points in the quantile-quantile-plot led to normally distributed error terms, but a slight negative correlation between random effects and error terms remained (r=-0.21, p=0.0053). Removing these five subjects from the dataset had only minor effects on the estimates of coefficients and on statistical significance.

Results

Supplementary table 1. Effect sizes and p-values for linear mixed models describing the stable isotope composition of crops as a function of the production system, year, and field replicate.

	$\delta^{15}N$		$\delta^{13}C$	
	Effect size (‰) Mean ± SE	p-value	Effect size (‰) Mean ± SE	p-value
System		<0.0001		0.96
OA vs. CON	+ 3.5 ± 0.3	<0.0001	+ 0.03 ± 0.10	0.77
OB vs. CON	+ 0.5 ± 0.3	0.050	+ 0.01 ± 0.10	0.90
OA vs. OB	+ 2.9 ± 0.3	<0.0001	- 0.02 ± 0.10	0.87
Year 2008 vs. 2007	+ 0.37 ± 0.22	0.10	+ 0.94 ± 0.08	<0.0001
Field replicate 2 vs. 1	- 0.19 ± 0.22	0.39	- 0.16 ± 0.08	0.056

$\delta^{15}N$: all crops except faba beans included in the model. $\delta^{13}C$: all crops included. SE = standard error. CON= conventional, OA and OB = organic fertilized with pig manure and legumes, respectively.

Supplementary table 2. Effect sizes and p-values for linear mixed models describing the diet's stable isotope composition as a function of production system, year, field replicate, and menu.

	$\delta^{15}N$		$\delta^{13}C$	
	Effect size (‰) Mean ± SE	p-value	Effect size (‰) Mean ± SE	p-value
System		<0.0001		0.12
OA vs. CON	+ 1.5 ± 0.2	<0.0001	+ 0.04 ± 0.07	0.57
OB vs. CON	+ 0.1 ± 0.2	0.56	+ 0.15 ± 0.07	0.070
OA vs. OB	+ 1.3 ± 0.2	<0.0001	-0.10 ± 0.07	0.18
Year 2008 vs. 2007	+ 0.05 ± 0.17	0.77	+ 0.78 ± 0.06	<0.0001
Field replicate 2 vs. 1	-0.02 ± 0.16	0.89	0.03 ± 0.06	0.74
Menu 2 vs. 1	-1.2 ± 0.2	<0.0001	- 0.24 ± 0.07	0.0009

Supplementary table 3. Effect sizes and p-values for linear mixed models describing the stable isotope composition in blood plasma as a function of production system, year, field replicate, and carryover.

	$\delta^{15}N$		$\delta^{13}C$	
	Effect size (‰) Mean ± SE	p-value	Effect size (‰) Mean ± SE	p-value
(system * day) interaction: difference of shift day 12 – day 0 for		<0.0001		0.0079
OA vs. CON	+ 0.27 ± 0.04	<0.0001	+ 0.04 ± 0.03	0.24
OB vs. CON	+ 0.05 ± 0.04	0.27	+ 0.10 ± 0.03	0.0024
OA vs. OB	+ 0.22 ± 0.04	<0.0001	- 0.06 ± 0.03	0.060
System (reference day 0)		0.0099		0.088
OA vs. CON	0.05 ± 0.03	0.13	+ 0.01 ± 0.04	0.73
OB vs. CON	0.00 ± 0.03	0.91	-0.07 ± 0.04	0.091
OA vs. OB	-0.06 ± 0.03	0.099	+ 0.09 ± 0.04	0.042
Year 2008 vs. 2007	+ 0.11 ± 0.09	0.19	0.44 ± 0.06	<0.0001
Field replicate 2 vs. 1	- 0.10 ± 0.09	0.24	-0.23 ± 0.06	0.0011
Day 12 vs day 0		<0.0001		<0.0001
Reference: system CON	- 0.19 ± 0.03	<0.0001	-0.35 ± 0.03	<0.0001
Reference: System OA	+ 0.08 ± 0.03	<0.0001	-0.31 ± 0.03	<0.0001
Reference: System OB	-0.14 ± 0.03	<0.0001	-0.25 ± 0.03	<0.0001
Carryover		0.0019		<0.0001
Any vs first intervention	range -0.07 to -0.12		range -0.08 to -0.15	

Stable isotope ratios as a function of production system x day interaction, production system, year, field replicate, day, and a carryover factor. The carryover factor has seven levels. A pairwise comparison of all these is not of interest, so only the study baseline is compared with all other levels. CON= conventional, OA and OB = organic fertilized with pig manure and legumes, resp. SE – standard error
